# A beta cell ATGL-lipolysis/adipose tissue axis controls energy homeostasis and body weight via insulin secretion in mice

**DOI:** 10.1007/s00125-016-4105-2

**Published:** 2016-09-27

**Authors:** Camille Attané, Marie-Line Peyot, Roxane Lussier, Pegah Poursharifi, Shangang Zhao, Dongwei Zhang, Johane Morin, Marco Pineda, Shupei Wang, Olivier Dumortier, Neil B. Ruderman, Grant A. Mitchell, Brigitte Simons, S. R. Murthy Madiraju, Erik Joly, Marc Prentki

**Affiliations:** 1 Montreal Diabetes Research Center, CRCHUM, 900 St-Denis (Viger Tower), Room R08-412, Montreal, QC H1W 4A4 Canada; 2 Present Address: UT Southwestern Medical Center, Dallas, TX USA; 3 Present Address: Diabetes Research Center, Beijing University of Chinese Medicine, Beijing, China; 4 Division of Medical Genetics, Department of Pediatrics, Université de Montréal and CHU Sainte-Justine, Montreal, QC Canada; 5 University Nice Sophia Antipolis, Nice, France; 6 Inserm, U1081, Institute for Research on Cancer and Aging of Nice (IRCAN), Aging and Diabetes Team, Nice, France; 7 CNRS, UMR7284, IRCAN, Nice, France; 8 Departments of Medicine and Physiology and Biophysics, Boston University School of Medicine, Boston, MA USA; 9 Diabetes Unit, Boston Medical Center, Boston, MA USA; 10 SCIEX, Concord, Ontario, Canada

**Keywords:** ATGL, Beta cell, Brown adipose tissue, Energy expenditure, High-fat diet fed mice, Insulin secretion, Insulin sensitivity, Lipolysis, Monoacylglycerol, White adipose tissue

## Abstract

**Aims/hypothesis:**

To directly assess the role of beta cell lipolysis in insulin secretion and whole-body energy homeostasis, inducible beta cell-specific adipose triglyceride lipase (ATGL)-deficient (B-*Atgl-*KO) mice were studied under normal diet (ND) and high-fat diet (HFD) conditions.

**Methods:**

*Atgl* ^flox/flox^ mice were cross-bred with *Mip-Cre*-ERT mice to generate *Mip-Cre*-ERT^/+^;*Atgl* ^flox/flox^ mice. At 8 weeks of age, these mice were injected with tamoxifen to induce deletion of beta cell-specific *Atgl* (also known as *Pnpla*2), and the mice were fed an ND or HFD.

**Results:**

ND-fed male B-*Atgl*-KO mice showed decreased insulinaemia and glucose-induced insulin secretion (GSIS) in vivo. Changes in GSIS correlated with the islet content of long-chain saturated monoacylglycerol (MAG) species that have been proposed to be metabolic coupling factors for insulin secretion. Exogenous MAGs restored GSIS in B-*Atgl*-KO islets. B-*Atgl*-KO male mice fed an HFD showed reduced insulinaemia, glycaemia in the fasted and fed states and after glucose challenge, as well as enhanced insulin sensitivity. Moreover, decreased insulinaemia in B-*Atgl-*KO mice was associated with increased energy expenditure, and lipid metabolism in brown (BAT) and white (WAT) adipose tissues, leading to reduced fat mass and body weight.

**Conclusions/interpretation:**

ATGL in beta cells regulates insulin secretion via the production of signalling MAGs. Decreased insulinaemia due to lowered GSIS protects B-*Atgl-*KO mice from diet-induced obesity, improves insulin sensitivity, increases lipid mobilisation from WAT and causes BAT activation. The results support the concept that fuel excess can drive obesity and diabetes via hyperinsulinaemia, and that an islet beta cell ATGL-lipolysis/adipose tissue axis controls energy homeostasis and body weight via insulin secretion.

**Electronic supplementary material:**

The online version of this article (doi:10.1007/s00125-016-4105-2) contains peer-reviewed but unedited supplementary material, which is available to authorised users.

## Introduction

In the aetiology of obesity-associated type 2 diabetes, hyperinsulinaemia is generally seen as a compensatory mechanism for insulin resistance induced by overfeeding. However, an alternative view is that fuel excess drives hyperinsulinaemia, which in turn results in obesity, insulin resistance followed by beta cell failure and diabetes [1–4]. Thus, reducing circulating insulin with diazoxide [5] or through partial ablation of the pancreas-specific *Ins1* gene [6, 7] protects from high-fat diet (HFD)-induced obesity and its associated complications. However, to our knowledge, it has never been shown that a subtle change specifically in a beta cell metabolic signalling pathway can modulate insulinaemia, energy homeostasis, body weight gain and the response to a high-energy diet.

Glucose and NEFA metabolism interface into the glycerolipid/NEFA (GL/NEFA) cycle, with its lipogenesis and lipolysis arms [8]. Lipolysis is mediated by the consecutive actions of adipose triglyceride lipase (ATGL) [9], catalysing the conversion of triacylglycerols (TGs) to diacylglycerols (DAGs), hormone-sensitive lipase (HSL) [10], hydrolysing DAG to monoacylglycerols (MAGs), and monoacylglycerol lipase [11, 12] and α/β-hydrolase domain-containing protein 6 (ABHD6) [13] hydrolysing MAGs to NEFA and glycerol. Lipolysis generates lipid signalling molecules for glucose-stimulated insulin secretion (GSIS) in beta cells [14], initial evidence coming from studies showing inhibition of GSIS in rat islets by the pan-lipase inhibitor orlistat [15] or an HSL inhibitor [16]. Whole-body and beta cell-specific HSL knockout (KO) mice showed a decreased insulin response to glucose in vivo and ex vivo [17, 18]. ATGL is the most abundant TG lipase in rat islets, and its production is regulated by nutritional status [19]. Whole-body *Atgl* (also known as *Pnpla*2) KO male mice are hypoinsulinaemic and hypoglycaemic, and show increased insulin sensitivity when fed a normal diet (ND) [19, 20] or HFD [21], as well as altered GSIS in vivo and ex vivo [19]. Moreover, we have recently shown that lipolysis-derived 1-MAG is a metabolic coupling factor for GSIS [13], confirming that beta cell lipolysis regulates GSIS.

In view of the importance of the GL/NEFA cycle in insulin secretion and protection from glucolipotoxicity [11], and as whole-body *Atgl-*KO mice show cardiomyopathy, TG steatosis in various tissues and lower plasma levels of TG and NEFA that influence insulin secretion, it is important to understand the role of ATGL specifically in the beta cells in the control of insulin secretion, in the protection of fuel surplus toxicity in vivo and in the regulation of whole-body energy homeostasis. Here we assessed the roles of beta cell ATGL in ND- and HFD-fed mice using conditional beta cell-specific *Atgl-*KO (B-*Atgl-*KO) mice.

## Methods

### Animals and metabolic in vivo studies

The breeding strategy is described in the electronic supplementary material (ESM) Methods. At 8 weeks of age, *Atgl* ^flox/flox^ (*fl/fl*), *Mip-Cre*-ERT (*MCre*) and *Mip-Cre*-ERT^/+^;*Atgl* ^flox^/^flox^ mice received daily intraperitoneal injections of tamoxifen as described in ESM Methods and were placed in individual cages. From 11 weeks of age, they were fed an ND (15% fat by energy; Harlan Teklad, Madison, WI, USA) or an HFD (Bio-Serv Diet no. F3282, 60% fat by energy; Frenchtown, NJ, USA) for 12 weeks. Body weight and food intake were measured weekly. All procedures were approved by the Institutional Committee for the Protection of Animals at the Centre Hospitalier de l’Université de Montréal.

Blood was collected between 08:00 and 10:00 hours in fed or overnight-fasted mice. Plasma glucose, insulin, NEFA, TG, glycerol and cholesterol were measured [17].

For the OGTTs, glucose (2 g/kg body weight) was administered orally in conscious mice at 09:00 hours after a 16 h fast. For the insulin tolerance tests (ITTs), insulin (0.75 U/kg body weight, Humulin; Lilly, Indianapolis, IN, USA) was injected intraperitoneally in conscious mice at 14:00 hours after 4 h of food withdrawal. OGTT and ITT were perfomed blinded.

Mice were placed in metabolic cages (Comprehensive Laboratory Animal Monitoring System, Columbus Instruments; Columbus, OH, USA) individually, for 3 days as previously described [22]. Metabolic mass was calculated as previously proposed [23].

Both male and female mice on a C57BL/6N background were used in this study. All animals were housed individually (after split in ND or HFD groups) at room temperature (23°C) with a 12 h light/12 h dark cycle. Mice were randomly distributed in either ND or HFD groups.

#### Western blotting

Proteins were extracted from isolated islets, hypothalamic nuclei and adipose tissue, and membranes were probed with antibodies against ATGL, phospho-HSL and total HSL, tubulin and β-actin as described in ESM Methods.

#### Insulin secretion ex vivo

Insulin secretion was measured in isolated islets immediately after isolation as previously described [24] with some modifications (see ESM Methods).

#### Islet metabolism

Measurement of lipolysis, glucose metabolism and oxygen consumption rate is described in ESM Methods.

#### Beta cell mass and pancreatic insulin content

Beta cell mass and pancreatic insulin content normalised by tissue weight were assessed as previously described [13, 25].

#### Intracellular Ca^2+^

Cytosolic Ca^2+^ was measured as previously described [24] with some modifications, detailed in ESM Methods.

#### Targeted lipidomics

GLs (MAGs, DAGs and TGs) were quantified by LC-MS/MS on lipid extracts from 250 islets incubated for 10 min in 3 or 16 mmol/l glucose as described in ESM Methods.

#### Adipose tissue metabolism

Lipolysis was measured on isolated adipocytes from visceral (perigonadal; VC) and subcutaneous (inguinal; SC) adipose tissues and interscapular brown adipose tissue (BAT) explants from 14-week-old male mice on an HFD (fed state) as previously described [26] with some modifications, detailed in ESM Methods.Fatty acid oxidation was measured as previously described [22].

#### RNA extraction and RT-PCR

Gene expression in adipose tissue was assessed as previously described [19] with some modifications (see ESM Methods and ESM Table 1).

#### Statistical analysis

Results are expressed as mean ± SEM. Statistical significance was calculated using the Student’s unpaired two-tailed *t* test or one-way or two-way ANOVA with Bonferroni post hoc test for multiple comparisons, as indicated using GraphPad Prism software (version 6.0, San Diego, CA, USA). A *p* value ≤0.05 was considered significant.

## Results

### Decreased insulinaemia and insulin response to glucose in B-*Atgl-*KO male mice on ND

**Fig. 1:**
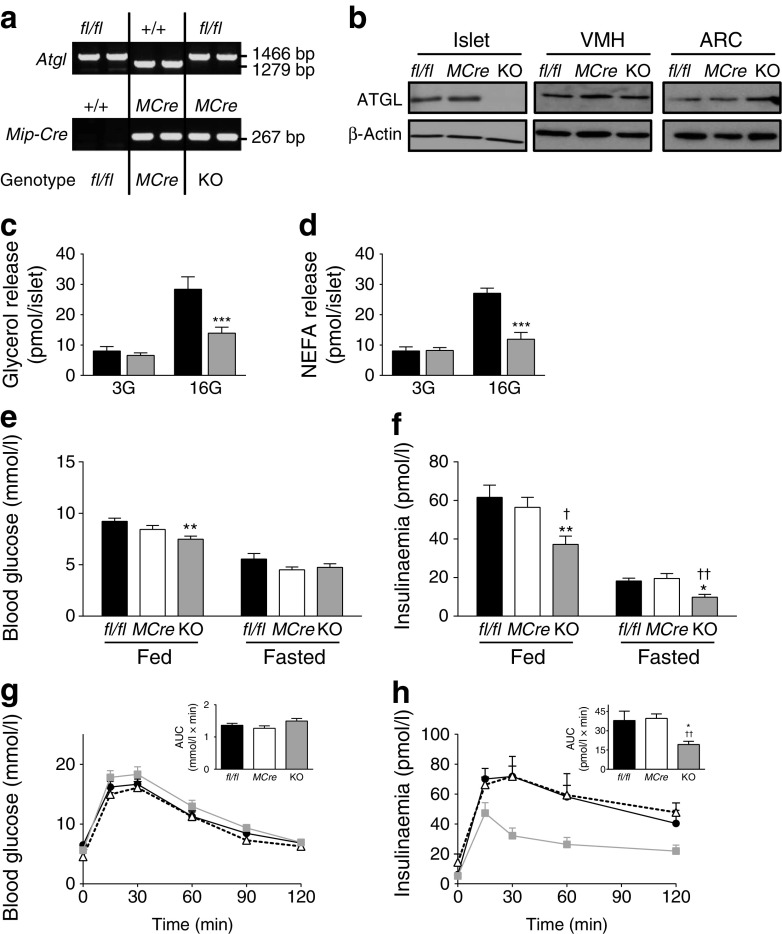
Reduced beta cell lipolysis, fed and fasted insulinaemia and GSIS in ND-fed B-*Atgl-*KO (KO) male mice. (**a**) Tail DNA from the offspring was used to determine the presence of the *Mip-Cre* transgene and to distinguish between normal (+/+) and homozygous (*fl/fl*) *Atgl* alleles. (**b**) ATGL protein production was measured by western blotting in extracts from isolated islets, VMH and ARC from 10-week-fasted mice. (**c**, **d**) Glycerol and NEFA released at 3 or 16 mmol/l glucose (3G or 16G) by isolated islets from *fl/fl* (black bars) or KO (grey bars) mice (*n* = 6 mice). Glycaemia and insulinaemia in *fl/fl* (black bars/circles), *MCre* (white bars/triangles) and KO (grey bars/squares) mice in fasted or fed states (**e**, **f**) or during OGTT (**g**, **h**). Insets depict AUC for glycaemia and insulinaemia. Data are mean ± SEM of 7–10 animals/group. *p < 0.05, **p < 0.01, ***p < 0.001 vs *fl/fl* and ^†^ *p* < 0.05, ^††^ *p* < 0.01 vs *MCre* (one-way ANOVA and Bonferroni post hoc test)

*fl/fl* mice were cross-bred with *MCre* mice producing Cre recombinase specifically in beta cells. The genotypes of the resulting offspring were ascertained for both the *Atgl* gene and *MCre* transgene (Fig. 1a). Eight-week-old *Mip-Cre*-ERT/*Atgl-*LoxP mice and control mice (*fl/fl* and *MCre* mice) were treated with tamoxifen to induce the deletion of the *Atgl* gene. Two weeks after tamoxifen treatment, ATGL protein production was markedly decreased (>90%) in islets from male B-*Atgl-*KO mice (Fig. 1b). No change of ATGL level was observed in the ventromedial hypothalamus (VMH) and arcuate nucleus (ARC) (Fig. 1b) or in liver and adipose tissue (not shown), indicating no leakage of Cre expression in these regions and confirming the tissue specificity of *MCre* transgene expression [27, 28]. Glucose-stimulated lipolysis was markedly decreased in islets from male B-*Atgl-*KO mice (Fig. 1c,d).

Ten-week-old male B-*Atgl-*KO mice on a ND displayed a modest but significant reduction in glycaemia only in the fed state (Fig. 1e), and reduced insulinaemia by about 50% (Fig. 1f) in both the fed and fasted states, when compared with control mice. An OGTT revealed a trend towards glucose intolerance (Fig. 1g) associated with reduced insulinaemia (approximately 60%) in male B-*Atgl-*KO mice (Fig. 1h). Body weight, pancreas weight, beta cell mass, insulin and protein content per islet and plasma TG, NEFA and glycerol were unchanged in B-*Atgl-*KO mice (ESM Table 2). Female B-*Atgl-*KO mice showed no differences in glycaemia and insulinaemia in fed and fasted states as well as during OGTT, compared with controls (ESM Fig. 1).

### Altered insulin secretion without changes in glucose metabolism and Ca^2+^ response in isolated islets from B-*Atgl-*KO islets

**Fig. 2:**
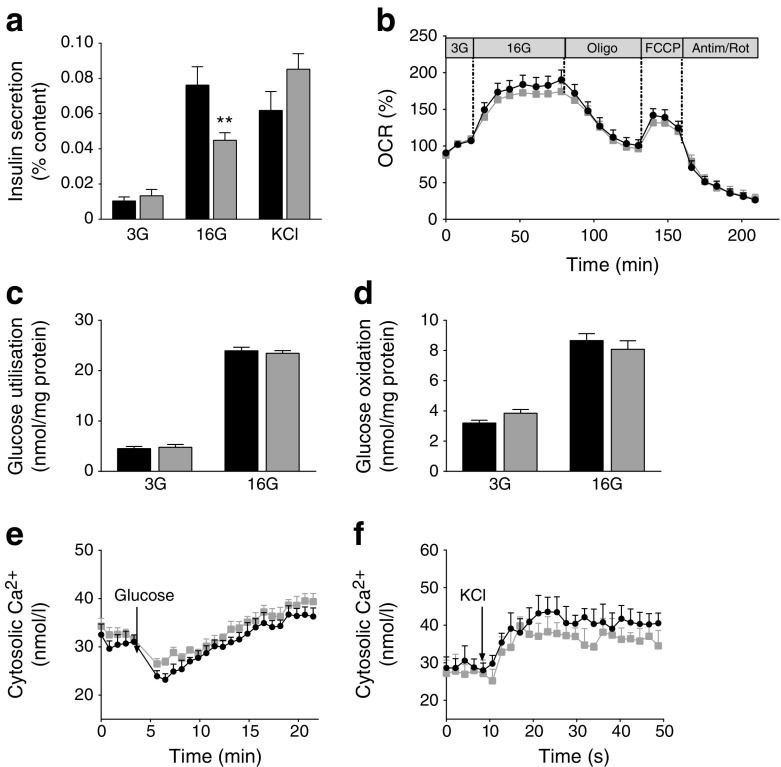
Reduced GSIS is not due to altered glucose and mitochondrial metabolism or cytosolic Ca^2+^ in isolated islets from B-*Atgl-*KO (KO) mice. (**a**) Insulin secretion in islets isolated from 10-week-old *fl/fl* (black bars) or KO (grey bars) mice incubated for 20 min at 3 or 16 mmol/l glucose (3G or 16G) and at 3 mmol/l glucose plus 35 mmol/l KCl (KCl) (*n* = 5–7 mice). (**b**) Oxygen consumption rate (OCR) of isolated islets from 10-week-old *fl/fl* (black circles) or KO (grey squares) mice at 3G (basal condition) or 16G and after the addition of 5 μmol/l oligomycin (Oligo), 1 μmol/l FCCP and 5 μmol/l rotenone/5 μmol/l antimycin (Antim/Rot). Glucose utilisation (**c**) and oxidation (**d**) in isolated islets from 10-week-old male mice at 3 and 16 mmol/l glucose (*n* = 6 mice). Cytosolic Ca^2+^ at 3G (basal condition) or in response to 16G glucose (**e**) or 35 mmol/l KCl (**f**) in dispersed islet cells (*n* = 5 mice). Data are mean ± SEM. **p < 0.01 vs *fl/fl* (Student’s *t* test)

Consistent with the altered insulin response to glucose observed in vivo, GSIS was markedly reduced in B-*Atgl-*KO islets, even though KCl-induced secretion was unchanged (Fig. 2a).

We then examined whether altered energy metabolism or calcium influx was contributing to the changes in insulin secretion response in the B-*Atgl-*KO islets. Oxygen consumption (coupled and uncoupled) (Fig. 2b) and glucose utilisation and oxidation (Fig. 2c,d) were unaffected in B-*Atgl-*KO islets. A similar rise in cytosolic Ca^2+^ levels in response to both glucose and KCl was observed in B-*Atgl-*KO and control islet cells (Fig. 2e,f). Thus, the altered GSIS observed in B-*Atgl-*KO islets cannot be explained by changes in glucose and mitochondrial metabolism or calcium influx.

### Reduced levels of specific saturated long-chain MAG species in B-*Atgl-*KO islets

**Fig. 3:**
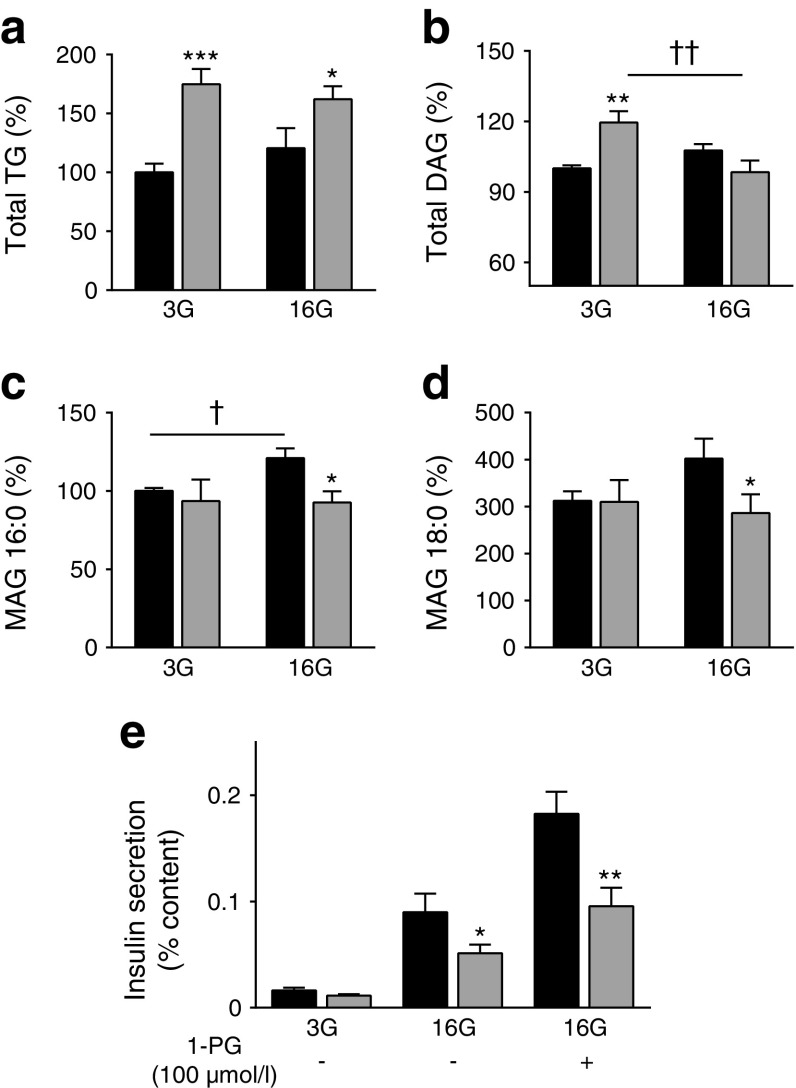
Reduced glucose-induced rise in saturated MAG species and restoration of insulin secretion by exogenous MAG in B-*Atgl-*KO (KO) islets. GL contents were measured in islets incubated for 10 min at 3 or 16 mmol/l glucose and the results expressed as a percentage of the control (3G *fl/fl*). (**a**) Total TG, (**b**) DAG, (**c**) MAG 16:0, and (**d**) MAG 18:0. (**e**) Insulin secretion in islets isolated from *fl/fl* (black bars) or KO (grey bars) incubated for 10 min at 3 or 16 mmol/l glucose ± 1-PG. Data are mean ± SEM. *p < 0.05, **p < 0.01, ***p < 0.001 vs *fl/fl* and ^†^ *p* < 0.05; ^††^ *p* < 0.01 3G vs 16G (Student’s *t* test)

We previously provided evidence that long-chain saturated 1-MAG species, in particular the relatively abundant 1-palmitoyglycerol (1-PG) (16:0) and 1-stearoylglycerol (18:0), act as coupling factors for GSIS [13]. As lipolysis is decreased in B-*Atgl-*KO islets, the formation of lipid signalling molecules such as DAG or MAG, which regulate insulin secretion, might be changed in these mice and affect GSIS. To address this, we used a targeted lipidomics approach to measure different GL species in isolated islets from *fl/fl* and B-*Atgl-*KO mice following incubation at low or high glucose concentration. As expected, total TG content was increased in B-*Atgl-*KO islets compared with *fl/fl* islets (Fig. 3a). Despite an increase at low glucose levels, total DAG content remained unchanged in the B-*Atgl-*KO islets with high glucose levels (Fig. 3b). Interestingly, 16:0 and 18:0 MAG species were increased at high glucose levels in control but not in B-*Atgl-*KO islets (Fig. 3c,d). We then examined whether the addition of 1-PG could rescue insulin secretion in B-*Atgl-*KO islets, showing that 1-PG was able to restore GSIS in B-*Atgl-*KO islets to the same level as in *fl/fl* islets without 1-PG. The extent of the increase in GSIS with exogenous 1-PG was similar (around twofold) in both B-*Atgl-*KO and *fl/fl* islets (Fig. 3e), even though the *fl/fl* islets showed higher levels of GSIS.

### Reduced body weight gain, insulin secretion and glycaemia and improved insulin sensitivity in B-*Atgl-*KO male mice fed an HFD

**Fig. 4:**
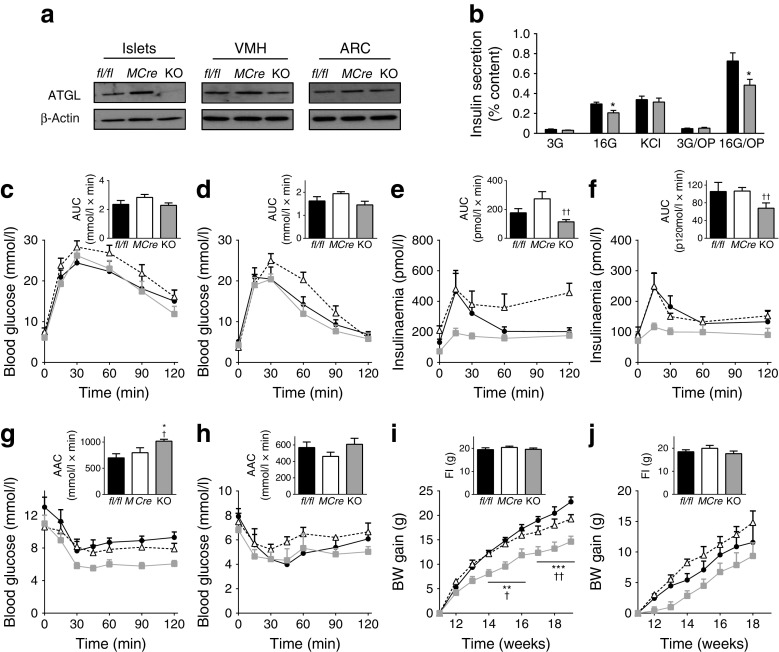
B-*Atgl-*KO (KO) male and female mice on HFD display reduced GSIS and body weight (BW) gain. (**a**) ATGL protein production in extracts of isolated islets, VMH or ARC of 23-week-old male mice fed an HFD. (**b**) Insulin secretion at 3 and 16 mmol/l glucose ± palmitate/oleate (OP; 0.15 mmol/l each) (3G/OP and 16G/OP) and at 3 mmol/l glucose plus 35 mmol/l KCl in isolated islets from 23-week-old male mice fed an HFD (*n* = 4–5 mice). Glycaemia and insulinaemia during OGTTs in 19-week-old male (**c**, **e**) or female (**d**, **f**) mice: black bars/circles, *fl/fl*; white bars/triangles, *MCre*; grey bars/squares, KO. Inset depicts AUC for glycaemia and insulinaemia curves. Glycaemia during an ITT in 21-week-old male (**g**) or female (**h**) mice. Inset depicts area above the curve (AAC). BW gain calculated relative to the weight prior to HFD feeding in male (**i**) and female (**j**) mice. Inset shows average food intake (FI). Data are mean ± SEM for 7–9 male and 4–5 female mice. *p < 0.05, **p < 0.01, ***p < 0.001 vs *fl/fl*; ^†^ *p* < 0.05 and ^††^ *p* < 0.01 vs *MCre* (one-way or two-way ANOVA and Bonferroni post hoc test)

Three weeks after tamoxifen treatment, mice were fed with either an ND or an HFD for 12 weeks. ATGL protein was undetectable in islets from 23-week-old B-*Atgl-*KO mice fed an HFD, with unaltered production in hypothalamic areas (Fig. 4a). Similar levels of plasma cholesterol, TG, NEFA and glycerol were observed in male and female B-*Atgl-*KO and control mice fed an HFD (Table 1). As observed in ND-fed mice, GSIS was decreased in B-*Atgl-*KO islets from male mice in the absence and presence of exogenous NEFA, whereas KCl-induced insulin secretion was not affected (Fig. 4b).

**Table 1:** Plasma variables of male and female mice fed an HFD

Variable	Male mice			Female mice		
	*fl/fl*	*MCre*	KO	*fl/fl*	*MCre*	KO
Fed glycaemia (mmol/l)	8.5 ± 0.4	7.7 ± 0.4	6.9 ± 0.4	6.5 ± 0.7	6.3 ± 0.6	6.9 ± 0.8
Fasted glycaemia (mmol/l)	7.4 ± 0.5	7.5 ± 1	6.0 ± 0.5	5.1 ± 0.5	4.6 ± 1	4.2 ± 0.8
Fed insulinaemia (pmol/l)	774 ± 189	1210 ± 287	365 ± 48*††	194 ± 29	361 ± 101	177 ± 63
Fasted insulinaemia (pmol/l)	120 ± 15	208 ± 30*	72 ± 10*††	89 ± 25	108 ± 20	70 ± 8
Fasted TG (mmol/l)	0.37 ± 0.03	0.46 ± 0.06	0.40 ± 0.05	0.29 ± 0.02	0.44 ± 0.05*	0.26 ± 0.03†
Fasted NEFA (mmol/l)	0.47 ± 0.04	0.37 ± 0.05	0.45 ± 0.05	0.61 ± 0.05	0.50 ± 0.05	0.46 ± 0.08
Fasted glycerol (mmol/l)	0.15 ± 0.01	0.19 ± 0.03	0.15 ± 0.02	0.29 ± 0.01	0.36 ± 0.02*	0.29 ± 0.02
Fasted FC (mmol/l)	0.73 ± 0.08	0.95 ± 0.11	0.75 ± 0.09	0.29 ± 0.06	0.40 ± 0.05	0.26 ± 0.01
Fasted CE (mmol/l)	2.41 ± 0.23	2.8 ± 0.22	2.39 ± 0.21	1.34 ± 0.19	1.59 ± 0.15	1.21 ± 0.17
Body weight (g)	52.9 ± 0.8	50.43 ± 1.1	45.7 ± 1.5**†	33.4 ± 3.2	38.1 ± 1.6	31.8 ± 3.2

Data are means ± SEM for 6–8 male and 4–5 female mice

Cholesterol ester (CE), free cholesterol (FC), TG, NEFA and glycerol were measured in plasma from anaesthetised, overnight-fasted male mice at 23 weeks of age. Glycaemia and insulinaemia were determined in 17-week-old fed or 19-week-old overnight-fasted conscious mice. Body weight was determined in 23-week-old mice

*p < 0.05, **p < 0.01 vs *fl/fl*; ^†^ *p* < 0.05, ^††^ *p* < 0.01 vs *MCre* (one-way ANOVA and Bonferroni post hoc test)

After 8 weeks on an HFD, glycaemia and insulinaemia were decreased in overnight-fasted and fed B-*Atgl-*KO male mice (Table 1). Even if no change in glucose tolerance was observed (Fig. 4c,d), plasma insulin levels were decreased during an OGTT in HFD-fed male (Fig. 4e) and female (Fig. 4f) KO mice. The reduced insulin secretion during OGTT in B-*Atgl-*KO mice with unaltered glucose tolerance suggests enhanced insulin sensitivity, which was confirmed by ITT in HFD-fed male mice (Fig. 4g) but not in HFD-fed female mice (Fig. 4h). B-*Atgl-*KO mice on an HFD showed decreased body weight gain compared with controls, without any change in food intake; this effect was already apparent after 3 weeks on the HFD and was more pronounced in male (Fig. 4i) than female (Fig. 4j) mice.

### Increased energy expenditure and adipose tissue metabolism in B-*Atgl-*KO male mice on an HFD

**Fig. 5:**
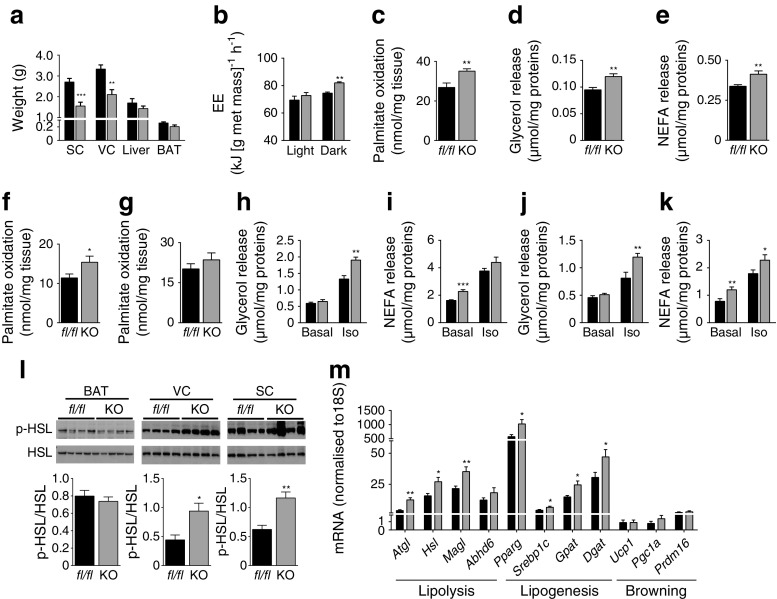
Increased whole-body EE and activation of WAT and BAT in B-*Atgl-*KO (KO) mice on an HFD. (**a**) Tissue weight of 23-week-old *fl/fl* (black bars) and KO (grey bars) male mice fed an HFD. (**b**) EE normalised by metabolic mass (met mass) in 14-week-old mice on an HFD. Palmitate oxidation and glycerol and NEFA release from BAT (**c–e**) Palmitate oxidation in VC (**f**) and SC (**g**); glycerol and NEFA release from VC (**h**, **i**) and SC (**j**, **k**) in 14-week-old mice on an HFD. Iso, Isoproterenol. (**l**) HSL phosphorylation on Ser563 in BAT, VC and SC and (**m**) gene expression in VC in 23-week-old mice on an HFD. Data are mean ± SEM for five mice. *p < 0.05, **p < 0.01, ***p < 0.001 (Student’s *t* test). *Srebp1c* is also known as *Srebf1*; *Gpat* is also known as *Gpam*; *Pgc1a* is also known as *Ppargc1a*

To understand how B-*Atgl-*KO mice are protected against diet-induced obesity, we measured whole-body energy expenditure (EE) and adipose tissue metabolism in HFD-fed male mice. As *MCre* and *fl/fl* mice display a similar phenotype on an HFD and the effects observed in B-*Atgl-*KO mice were less pronounced in female than male mice, the rest of the experiments were carried out only with male mice, using *fl/fl* mice as a control. VC and SC weight decreased in B-*Atgl-*KO mice compared with *fl/fl* mice, while liver and BAT weight did not differ (Fig. 5a). After 48 h acclimatisation in metabolism cages, EE was measured in 14-week-old B-*Atgl-*KO mice that had been on an HFD for only 3 weeks, before any significant change in body weight had occurred. EE was increased in B-*Atgl-*KO mice during the dark period without a change during the light period (Fig. 5b). As BAT plays a major role in the regulation of whole-body EE, we measured BAT metabolism ex vivo. Palmitate oxidation and lipolysis were higher in BAT explants from B-*Atgl-*KO mice (Fig. 5c–e). Moreover, palmitate oxidation was increased in VC (Fig. 5f) but not in SC (Fig. 5g) from B-*Atgl-*KO mice, which could also contribute to the observed enhanced EE in B-*Atgl-*KO (Fig. 5b).

As B-*Atgl-*KO mice displayed decreased insulinaemia and since insulin inhibits lipolysis in white adipose tissue (WAT), we then assessed whether basal and isoproterenol-stimulated lipolysis were changed in isolated adipocytes from VC and SC. Interestingly, isoproterenol-stimulated lipolysis and basal NEFA release were increased in B-*Atgl-*KO adipocytes from VC and SC (Fig. 5h–k). Levels of HSL Ser563 phosphorylation in VC and SC were consistently increased in overnight-fasted B-*Atgl-*KO mice (Fig. 5l). Finally, an increased expression of genes involved in both lipolysis and lipogenesis pathways was observed in VC (Fig. 5m) but not in SC or BAT (not shown) from B-*Atgl-*KO mice without changes in browning-related gene expression, suggesting an increase in GL/NEFA cycle activity in VC. Overall, the decreased body weight gain observed in B-*Atgl-*KO mice can be explained by increased whole-body EE, activation of BAT and VC and SC lipolysis and fat oxidation, and enhanced futile (energy-consuming) GL/NEFA cycling in VC.

## Discussion

The importance of ATGL as the major TG hydrolase during lipolysis is well known, and we have previously provided evidence indicating the regulatory role of lipolysis in beta cells in insulin secretion regulation [14]. Even though the earlier work showed that global deletion of ATGL affects whole-body glucose and lipid metabolism [9, 10] and GSIS [19], the specific role of beta cell ATGL and lipolysis in whole-body energy homeostasis, insulin secretion and action, and adipose tissue function is not known. Employing conditional B-*Atgl-*KO mice, we now demonstrate that beta cell ATGL is important in glucose signalling for insulin secretion via the production of saturated long-chain 1-MAG, a lipid metabolic coupling factor for the amplification of GSIS [13, 24]. Our results also show that deletion of ATGL specifically in beta cells is, by lowering insulin secretion response, able to protect against HFD-induced hyperinsulinaemia, insulin resistance and obesity, and also able to promote elevated EE by activating BAT, and lipolysis and fat oxidation in WAT.

Altered GSIS in B-*Atgl-*KO islets is associated with a decreased content of saturated MAG species and is restored by exogenous 1-MAG, supporting the view that ATGL controls the production of 1-MAG acting as a coupling factor for GSIS. In contrast to a recent beta cell *Atgl-*KO study [29] that will be discussed below, we did not find any evidence that ATGL deletion in beta cells is associated with an alteration in beta cell glucose and mitochondrial metabolism. Additionally, the glucose-induced Ca^2+^ rise and KCl-induced insulin secretion were also unchanged in the B-*Atgl-*KO islets. Thus, the reduction in GSIS in B-*Atgl-*KO islets is not due to alterations in energy metabolism or Ca^2+^ signalling, but can be explained, at least in part, by a decreased content of lipolysis-derived MAG species.

We noticed sex-dependent differences in insulin secretion response to diet in the B-*Atgl-*KO mice. Thus, male, but not female, B-*Atgl-*KO on the ND displayed reduced GSIS in vivo, whereas both male and female B-*Atgl-*KO mice showed reduced GSIS with an HFD. This proves the general importance of ATGL for GSIS, and the compensatory mechanisms involving other lipases (e.g. HSL and carboxylesterases) probably override the consequences of ATGL deletion in female mice on a ND.

**Fig. 6:**
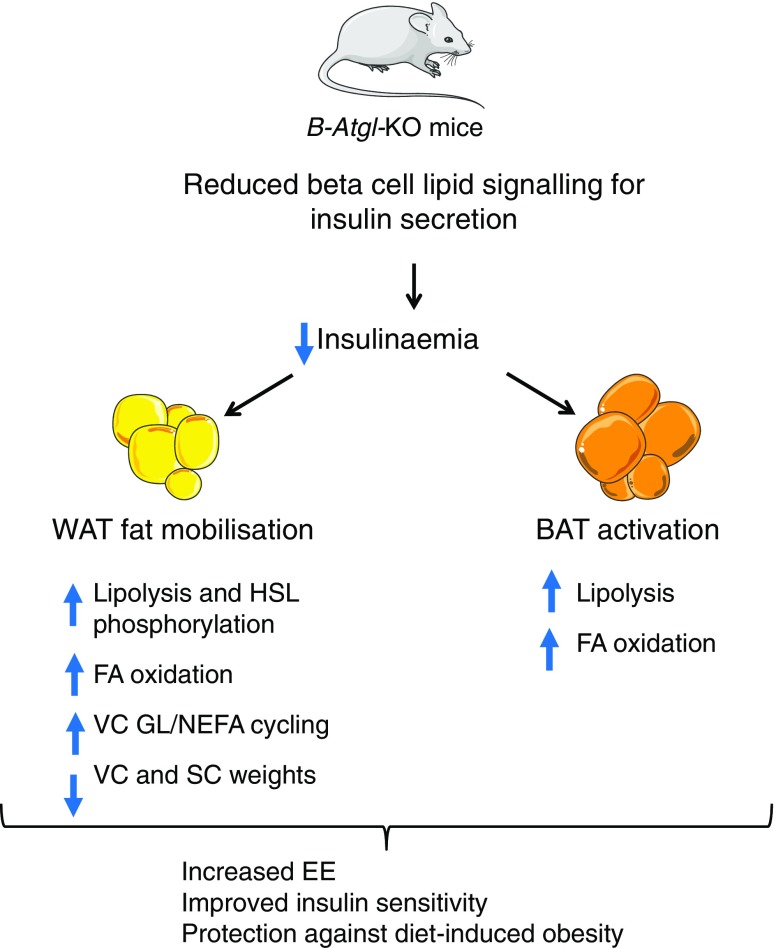
Model depicting how an islet beta cell ATGL-lipolysis/adipose tissue axis controls energy homeostasis and body weight via insulin secretion. Deletion of *Atgl* specifically in beta cells affects saturated MAG content, leading to altered lipid signalling for insulin secretion. Under HFD conditions, circulating insulin is decreased in B-*Atgl-*KO mice, leading to: (1) increased lipolysis, NEFA oxidation and the expression of genes involved in GL/NEFA cycling in VC; (2) increased lipolysis in SC; and (3) increased lipolysis and fat oxidation in BAT. Overall, WAT fat mobilisation, enhanced futile GL/NEFA cycling in VC and BAT activation contribute to increased EE, reduced fat storage, improved insulin sensitivity and protection against diet-induced obesity. FA, fatty acid

In as much as *Atgl* deletion only in beta cells is able to protect against obesity-associated complications, this study brings out a new and previously unrecognised role for beta cell ATGL and associated lipolysis-derived GSIS-promoting signal(s) in the control of whole-body energy, insulin and glucose homeostasis, and the development of obesity. Thus, decreased insulinaemia in B-*Atgl-*KO male mice on an HFD leads to increased EE, improved insulin sensitivity and decreased body weight gain on an HFD via WAT fat mobilisation and BAT activation (Fig. 6). Our results are in line with studies showing that reducing circulating insulin with diazoxide [5], partial ablation of the *Ins1* gene [6, 7] or the inhibition of insulin signalling by genetic or pharmacological approaches [30, 31] protects from obesity and the metabolic syndrome. In these studies, protection against diet-induced obesity has been explained by increased EE due in part to increased BAT activity and browning of WAT [6, 30, 31]. In the present study, we observed that increased lipolysis in VC and SC is associated with increased HSL phosphorylation and also the production of different lipases in VC. Interestingly, the levels of transcription factors and enzymes involved in lipogenesis were also enhanced in the VC of B-*Atgl-*KO male mice, suggesting an increase in futile GL/NEFA cycling [11] coupled with enhanced NEFA β-oxidation, which can also contribute to the increased EE and protection against diet-induced obesity. Taken together, these results support the concept that an islet beta cell ATGL–lipolysis/adipose tissues axis controls energy homeostasis and body weight via insulin secretion and insulinaemia.

The current results are at marked variance with a recent study by Tang et al [29] that examined the consequences of *Atgl* deletion on beta cell function specifically in beta cells under HFD conditions. Contrary to our results, the B-*Atgl-*KO male mice Tang et al generated showed unchanged body weight gain and insulin sensitivity, an almost total abolition of GSIS in vivo, increased glycaemia in the fed state and marked glucose intolerance. The isolated islets showed alterations in oxygen consumption and mitochondrial function. These discrepancies can be explained by three main differences between the two studies. First, the HFD model used is different. In Tang et al’s paper, experiments were performed on 8-week-old male mice with 4 weeks’ HFD feeding directly after weaning, a rarely used protocol, whereas we used 23-week-old mice with 12 weeks of HFD feeding from 11 weeks of age. The second difference is in the Cre recombinase transgenic mice used to create the beta cell-specific gene deletion. To avoid compensatory mechanisms in vivo that can occur when a protein is deleted during development, we used an inducible conditional *Atgl-*KO in adult beta cells. In contrast, Tang et al mainly used Rip-Cre mice in which ATGL is deleted during development. Third, the B-*Atgl-*KO mice were bred from different C57BL/6 backgrounds: C57BL/6N in our study vs C57BL/6J in Tang et al’s study. Great concerns exist regarding the use of mice on a C57BL/6J background for beta cell studies. Indeed, this strain shows altered insulin secretion and expresses a mutation in nicotinamide nucleotide transhydrogenase, an enzyme involved in mitochondrial energy metabolism, NADPH production and the regulation of GSIS [14, 25, 32, 33]. Overall, strain background, the *Cre*-expressing mouse model (promoter and age of deletion) and the HFD protocol can explain the discrepancies observed between our results and those of Tang et al.

It has recently been shown that *MCre* mice express a human growth hormone (hGH) minigene in pancreatic islets, and that local hGH secretion is associated with increased insulin content and resistance to streptozotocin-induced hyperglycaemia [34]. However, *MCre* mice exhibited normal glycaemia, glucose tolerance and insulin sensitivity and appropriate GSIS [34]. In our study, even if hGH is present on the transgene construct used to generate the *MCre* mice [28], it does not create a specific phenotype in *MCre* mice compared with *fl/fl* mice for all the variables that we studied.

In summary, our results demonstrate that beta cell ATGL regulates GSIS via the production of lipolysis-derived long-chain saturated MAG, a metabolic coupling factor for insulin secretion. Modulation of lipolysis in beta cells by ATGL regulates whole-body energy homeostasis in the context of metabolic stress, by preventing exacerbated insulin secretion. Indeed, decreased insulinaemia leads to BAT activation and WAT fat mobilisation to increase EE, improve insulin sensitivity and protect against HFD-induced obesity. These results support the concept that fuel excess drives obesity and diabetes via hyperinsulinaemia and provide an additional impetus to lower insulin hypersecretion as a potential therapeutic approach for obesity and diabetes.

## Electronic supplementary material

Below is the link to the electronic supplementary material.

ESM 1 (PDF 401 kb)

## Abbreviations

1-PG: 1-Palmitoylglycerol
ARC: Arcuate nucleus
ATGL: Adipose triglyceride lipase
BAT: Brown adipose tissue
B-*Atgl-*KO: Beta cell-specific *Atgl-*KO
DAG: Diacylglycerol
*fl/fl*: *Atgl* ^flox/flox^
GL: Glycerolipid
GSIS: Glucose-stimulated insulin secretion
HFD: High-fat diet
hGH: Human growth hormone
HSL: Hormone-sensitive lipase
ITT: Insulin tolerance test
KO: Knockout
MAG: Monoacylglycerol
*MCre*: *Mip-Cre*-ERT
ND: Normal diet
SC: Subcutaneous adipose tissue
TG: Triacylglycerol
VC: Visceral adipose tissue
VMH: Ventromedial hypothalamus
WAT: White adipose tissue
