# Supplementary material for: A beta cell ATGL-lipolysis/adipose tissue axis controls energy homeostasis and body weight via insulin secretion in mice
Source: Diabetologia. 2016 Sep 27;59(12):2654–63. doi: 10.1007/s00125-016-4105-2 (PMC6518076; doi:10.1007/s00125-016-4105-2)
Supplement: Supplementary file 1 — (PDF 401 kb) [file 125_2016_4105_MOESM1_ESM.pdf]

ESM Methods

Breeding strategy

Homozygous *Atgl* flox/flox mice (*fl/fl*) in which exon 1 of *Pnpla2* (*Atgl*) gene is flanked with LoxP sites were generated as previously described [1] and were backcrossed for 6 generations with C57BL/6J mice (Jackson Laboratory, Bar Harbor, ME, USA). Heterozygous *Mip* (murine Ins1 promoter)-*Cre*-ERT mice (*MCre*) were generated as previously described [2] and were backcrossed for 6 generations with C57BL/6N mice (Charles River, Saint Constant, QC, Canada). *MCre* mice were first crossed with *fl/fl* mice and *MCre*/+; *Atgl* flox/+ and +/+; *Atgl* flox/+ mice obtained were then bred to produce *MCre* /+; *Atgl* flox/flox mice. Offspring tail DNA was used to confirm the presence of *Atgl* gene by the specific amplification of a 1466 bp DNA fragment in *fl/fl* mice, a 1279 bp fragment in the +/+ mice and the presence of the *MCre* transgene by a 267 bp DNA fragment in the *MCre* mice [1, 2].

As *fl/fl* mice were on C57BL/6J background, we ascertained by PCR that none of the mice used in this study carried the mutation in the nicotinamide nucleotide transhydrogenase gene reported in the C57BL/6J strain [3]. All procedures were approved by the Institutional Committee for the Protection of Animals at the Centre Hospitalier de l'Université de Montréal. At 8 weeks of age, *fl/fl*, *MCre* and *MCre*/+; *Atgl* flox/flox mice received daily intraperitoneal injections of tamoxifen (50 mg/kg body weight, dissolved in 90% corn oil plus 10% ethanol) for 5 consecutive days) to induce Cre recombinase and the deletion of *Atgl* in the *MCre*/+; *Atgl* flox/flox mice (B-*Atgl* -KO).

Western blot

Proteins were extracted as described previously [4]. For adipose tissue samples, homogenates were centrifuged at 10, 000 g for 10 min at 4°C and infranatant was collected to quantify proteins. Antibodies and dilutions are listed below.

Protein	Provider	Reference	Dilution
ATGL	Cell Signaling	2138	1/1000
beta-actin	Sigma Aldrich	A5441	1/5000
Phospho-HSL (Ser563)	Cell Signaling	4139S	1/1000
Total HSL	Cell Signaling	4107	1/1000
Tubulin	Abcam	ab 4074	1/10000

Insulin secretion ex-vivo

Islets were distributed in 12-well plates (10 islets/well) in RPMI medium containing 3 mmol/l glucose for 2 h and preincubated for 45 min at 37°C in KRB medium with 10 mmol/l Hepes (KRBH) containing 0.5% defatted-BSA and 3 mmol/l glucose. Islets were then incubated for 20 min at 3 or 16 mmol/l glucose and at 3 mmol/l glucose plus 35 mmol/l KCl. For experiments on isolated islets from 23-week-old HFD-fed mice, islets were incubated for 1 h at 3 or 16 mmol/l glucose, in the presence or absence of palmitate/oleate (0.15 mmol/l each). For rescue experiment with 1-PG, islets were incubated in the presence or absence of 100 µmol/l 1-PG during the pre-incubation at 3 mmol/l glucose and the incubation at 3 or 16 mmol/l glucose. Stock solution of 1-PG was prepared in DMSO. Each condition was run in 3 replicates. Insulin release was normalized for the total islet insulin content.

Islet metabolism

Lipolysis was measured on 100 islets isolated from 10-week-old male mice and incubated for 1h in KRBH 0.5% defatted-BSA at 3 or 16 mmol/l glucose. At the end of the incubation, media were kept to measure glycerol [5] and NEFA [6] release.

Glucose oxidation and utilization were assessed as described previously on islets isolated from 10-week-old male mice [4].

Oxygen consumption rate (OCR) was measured using a Seahorse XF24 Analyzer at 3 and 16 mmol/l glucose, in the absence or presence of 5 µmol/l oligomycin, 1 µmol/l FCCP and 5 µmol/l rotenone plus 5 µmol/l antimycin on islets isolated from 10-week-old male mice [7].

Intracellular Ca^{2+}

After isolation, islets were dispersed into single cells by trypsin digestion [8] and were seeded at a density of 60,000 cells per well in 96-well black plates with clear bottom coated with extracellular matrix derived from 804G cells (804G-ECM; gift from Philippe Halban, Geneva, Switzerland) [9]. After 3h incubation in RPMI medium containing 6 mmol/l glucose and 10% FBS to allow cell attachment, cells were loaded with Fura-2 AM (6 µmol/l) dissolved in pluronic F-127 for 75 min and calcium was measured as previously described [10]. Each condition was run in 5 replicates. Cytosolic calcium was calculated according to Grynkiewicz et al. [11].

Targeted lipidomics Batches of 250 islets were incubated for 10 min in KRBH, 0.5% defatted-BSA at 3 or 16 mmol/l glucose. At the end of the incubation, islets were washed

twice in cold PBS, transferred in glass tubes containing methanol and 0.02% of 2,6-di-tert-butyl-4-methylphenol and stored at -80°C. 100µl synthetic lipid standard mix at 0.1 µmol/l (d5-MAG 1-18:1, DAG 17:0/17:0 and TG 19:0/12:0/19:0) was added and lipid extraction was done using Folch method [12]. Dried lipids were reconstituted in 150µL of chloroform/methanol (1:2) plus 5 mmol/l ammonium acetate (AmAc) and injections of 20µl were performed on a LC–MS/MS system composed of a Shimadzu Prominence XR UFPLC coupled to a TripleTOF 6600 mass spectrometer (SCIEX) operated in positive electrospray ionization mode. Lipids were separated on an Atlantis dc18 column (2.1×50 mm, 3 µm particle size) (Waters). The mobile phase consisted of solvent A (45% H_2O, 35% acetonitrile and 20% methanol with 5 mmol/l AmAc) and solvent B (80% acetonitrile and 20% methanol with 5 mmol/l AmAc). Targeted MS/MS lists, with optimized collision energies were designed for each lipid species and included retention times for all the major MAG, DAG and TG in order to confirm the lipid composition and extract a specific set of fragment-derived chromatograms (XIC) using high mass accuracy (m/z value± 0.01Da) for lipid quantification by LC peak area integration. Quantification was performed by integrating peak XIC areas from the selected precursor and fragment ion sets using Multiquant software (version 3.0.2; SCIEX) and normalized by corresponding internal standard peak areas. Total MAG, DAG and TG were determined as the sum of the corresponding extracted ion chromatographic peak areas.

Adipose tissue metabolism

For lipolysis, isolated adipocytes were prepared from visceral (perigonadal) and subcutaneous (inguinal) adipose tissues (VC and SC respectively). Briefly, fat tissues were

minced and digested in a KRBH (pH 7.4) supplemented with 2% fatty acid–free BSA and 1 mg/mL type I collagenase (Sigma- C6885) at 37°C for 30 min under shaking. Adipocyte suspension was then washed two times and cells were counted and resuspended in KRBH supplemented with 4 mmol/l glucose, 1 unit/mL adenosine deaminase and 2% fatty acid–free BSA (lipolysis buffer). BAT was minced in small pieces (~1 mm) and explants were washed two times with KRBH supplemented with 2% fatty acid–free BSA. 50µl of adipocyte suspension (around 50,000 cells) or 50 mg of BAT explants were added to 450 µl lipolysis buffer, in the presence or the absence of 1 µmol/l isoproterenol (Sigma I6504). After 2 h incubation at 37°C under gentle shaking, 200 µL of infranatant was removed and kept to measure glycerol and NEFA release using commercial kits (Sigma- F6428 and Wako diagnostic NEFA-HR, respectively).

RNA extraction and RT-PCR

Adipose tissue (100mg) was homogenized in 1 ml Qiazol (Qiagen) and 200µl chloroform were added. Samples were centrifuged at 10000 g for 15 minutes at 4°C and upper phase was collected and mixed with 1 volume Ethanol 70%. RNA extraction was then continued using RNeasy Mini Kit (Qiagen) following kit protocol. 2 µg RNA were reverse-transcribed to cDNA and gene expression was determined by the standard curve method and normalized to the expression of 18S as described previously [4]. The primers (IDT, Coralville, IA) used are listed in ESM Table 1.

ESM Table

ESM Table 1: Primer sequence

Ucp1	Forward	CTT TGC CTC ACT CAG GAT TGG
	Reverse	ACT GCC ACA CCT CCA GTC ATT
Pparg	Forward	GGT CAG CTC TTG TGA ATG GAA
	Reverse	ATC AGC TCT GTG GAC CTC TCC
Pgc1a	Forward	TAG AGT GTG CTG CTC TGG TTG
	Reverse	GAT TGG TCG CTA CAC CAC TTC
Prdm16	Forward	CAG CAC GGT GAA GCC ATT C
	Reverse	GCG TGC ATC CGC TTG TG
Srebp1	Forward	ATG CTC CAG CTC ATC AAC AAC
	Reverse	GAG GCC AGA GAA GCA GAA GAG
Atgl	Forward	TCCCACTTTAGCTCCAAGGAT
	Reverse	AGCTTCCTCTGCATCCTCTTC
Hsl	Forward	GGC TCA CAG TTA CCA TCT CAC C
	Reverse	GAG TAC CTT GCT GTC CTG TCC
Magl	Forward	GTGCCTACCTGCTCATGGAAT
	Reverse	GAGGACGGAGTTGGTCACTTC
Abhd6	Forward	AGACCAGGTGCTTGATGT
	Reverse	CTCTCCATCACTACCGAAT
Gpat	Forward	CGG AAC TGA ACT GGA GAA GTG
	Reverse	GAT GAA TTG CTG GTG CTC CTT
Dgat1	Forward	GAG CTA TCC AGA CAA CCT GAC C
	Reverse	AGC ATC TCA AGA ACT CGT CGT
18s	Forward	CTG AGA AAC GGC TAC CAC ATC
	Reverse	GGC CTC GAA AGA GTC CTG TAT

ESM Table 2: Characteristics and blood chemistry of male mice fed a ND.

	fl/fl	*MCre*	**KO**
**Body Weight (g)**	27.9 ± 0.6 (12)	29.4 ± 0.5 (9)	28.2 ± 0.3 (15)
**Pancreas Weight (mg)**	205 ± 9 (5)	255 ± 11 (4)	234 ± 9 (6)
**Beta Cell Mass (mg)**	1.65 ± 0.27 (5)	1.67 ± 0.30 (5)	2.20 ± 0.32 (5)
**Insulin Content/Pancreas (ng/mg)**	137 ± 13 (5)	171 ± 14 (6)	198 ± 16 (4) *
**Insulin Content/islet (ng)**	39.0 ± 2.5 (10)	43.9 ± 4.4 (6)	37.8 ± 1.7 (9)
**Protein Content/islet (µg)**	0.358 ± 0.037 (9)	0.388 ± 0.050 (6)	0.413 ± 0.023 (9)
**Fed TG (mmol/l)**	0.34 ± 0.03 (10)	0.40 ± 0.03 (9)	0.35 ± 0.02 (11)
**Fasted TG (mmol/l)**	0.26 ± 0.03 (7)	0.29 ± 0.03 (8)	0.21 ± 0.01 (7)
**Fed NEFA (mmol/l)**	0.17 ±0.02 (10)	0.19 ± 0.01 (9)	0.17 ± 0.02 (11)
**Fasted NEFA (mmol/l)**	0.39 ± 0.09 (7)	0.38 ± 0.05 (8)	0.34 ± 0.04 (7)
**Fasted Glycerol (mmol/l)**	0.29 ± 0.05 (6)	0.32 ± 0.02 (6)	0.23 ± 0.02 (6)

Means ± SEM of n animals as indicated in parentheses.

All the variables were determined in 10-week-old male mice (2 weeks after tamoxifen treatment) fed a ND. Triacylglycerol (TG), non-esterified fatty acids (NEFA), and glycerol were measured in plasma from anesthetized overnight fasted and fed male mice. *$p<0.05$ vs *fl/fl* (one-way ANOVA and Bonferroni post hoc test).

ESM Fig. 1

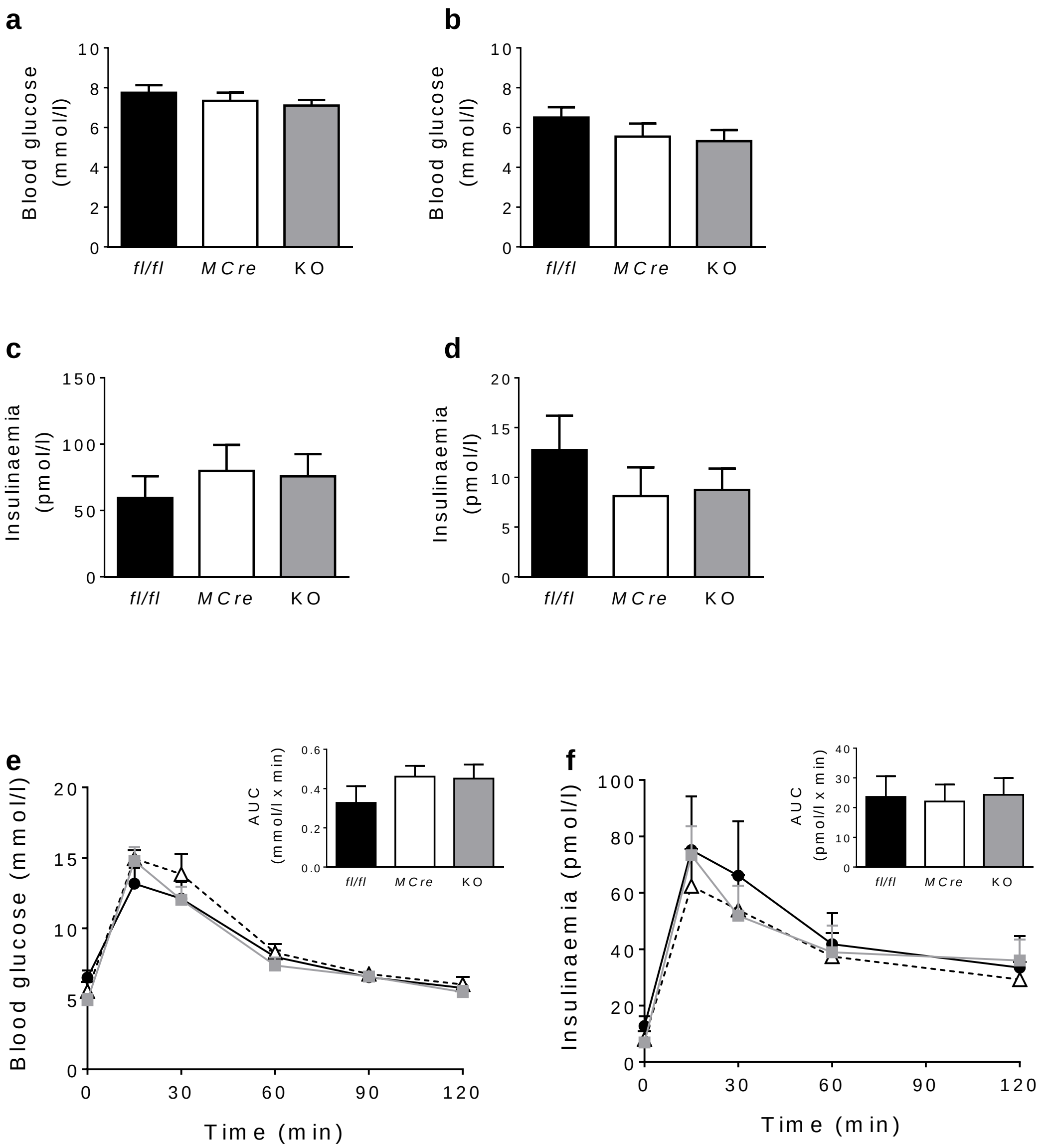

ESM Fig. 1: Insulinaemia and glucose-induced insulin secretion are unchanged in B-ATGL-KO female mice fed a ND. Glycaemia **(a, b)** and insulinaemia (**c, d)** were measured in fed **(a, c)** or overnight fasted **(b, d)** *fl/fl* (black bar), *MCre* (white bar) or KO (grey bar) female mice at 10 weeks of age. Glycaemia **(e)** and insulinaemia **(f)** during an OGTT in 10-week-old *fl/fl* (black bars/circles), *MCre* (white bars/triangles) or KO mice (grey bars/squares) after overnight fasting. Inset depicts AUC for glycaemia and insulinaemia. Means +/- SEM of 5 to 8 animals/group.

ESM - References

1. Wu JW, Wang SP, Alvarez F, et al (2011) Deficiency of liver adipose triglyceride lipase in mice causes progressive hepatic steatosis. Hepatology 54:122–132.

2. Tamarina NA., Roe MW, Philipson LH (2014) Characterization of mice expressing Ins1 gene promoter driven CreERT recombinase for conditional gene deletion in pancreatic ??-cells. Islets 6:37–41.

3. Fergusson G, Éthier M, Guévremont M, et al (2014) Defective insulin secretory response to intravenous glucose in C57Bl/6J compared to C57Bl/6N mice. Mol Metab 3:848–854.

4. Peyot M-L, Guay C, Latour MG, et al (2009) Adipose triglyceride lipase is implicated in fuel- and non-fuel-stimulated insulin secretion. J Biol Chem 284:16848–16859.

5. Bradley DC, Kaslow HR (1989) Radiometric assays for glycerol, glucose, and glycogen. Anal Biochem 180:11–16.

6. Zhao S, Mugabo Y, Iglesias J, et al (2014) α/β-Hydrolase Domain-6-accessible monoacylglycerol controls glucose-stimulated insulin secretion. Cell Metab 19:993–1007.

7. Pepin É, Al-mass A, Attané C, et al (2016) Pancreatic β -Cell Dysfunction in Diet-Induced Obese Mice : Roles of AMP-Kinase , Protein Kinase C ε , Mitochondrial and Cholesterol Metabolism, and Alterations in Gene Expression. PLoS One 11:1–18.

8. Peyot M-L, Pepin E, Lamontagne J, et al (2010) Beta-cell failure in diet-induced obese mice stratified according to body weight gain: secretory dysfunction and altered islet lipid metabolism without steatosis or reduced beta-cell mass. Diabetes 59:2178–87.

9. Dumortier O, Hinault C, Gautier N, et al (2014) Maternal protein restriction leads to pancreatic failure in offspring: role of misexpressed microRNA-375. Diabetes 63:3416–27.

10. Zhao S, Poursharafi P, Mugabo Y, et al (2015) α/β-Hydrolase Domain-6 and Saturated Long Chain Monoacylglycerol Regulate Insulin Secretion Promoted By Both Fuel and Non-Fuel Stimuli. Mol Metab 4:940-50.

11. Grynkiewicz G, Poenie M, Tsien RY (1985) A new generation of Ca2+ indicators with greatly improved fluorescence properties. J Biol Chem 260:3440–3450.

12. Folch J, Lees M, Sloane Stanley GH (1957) A simple method for the isolation and purification of total lipides from animal tissues. J Biol Chem 226:497–509.
